# Protease Activity Analysis: A Toolkit for Analyzing Enzyme Activity Data

**DOI:** 10.1021/acsomega.2c01559

**Published:** 2022-07-06

**Authors:** Ava P. Soleimany, Carmen Martin-Alonso, Melodi Anahtar, Cathy S. Wang, Sangeeta N. Bhatia

**Affiliations:** † Harvard-MIT Division of Health Sciences and Technology, MIT, Cambridge, Massachusetts 02139, United States; ‡ Program in Biophysics, Harvard University, Boston, Massachusetts 02115, United States; § Microsoft Research New England, Cambridge, Massachusetts 02142, United States; ∥ Department of Biological Engineering, MIT, Cambridge, Massachusetts 02139, United States; ⊥ Department of Electrical Engineering and Computer Science, MIT, Cambridge, Massachusetts 02139, United States; # Howard Hughes Medical Institute, Cambridge, Massachusetts 02139, United States

## Abstract

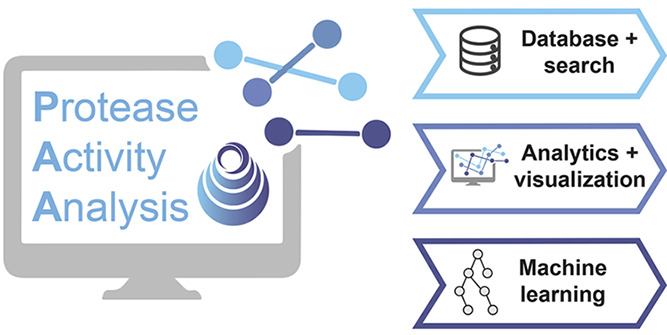

Analyzing the activity of proteases and their substrates is critical to defining the biological functions of these enzymes and to designing new diagnostics and therapeutics that target protease dysregulation in disease. While a wide range of databases and algorithms have been created to better predict protease cleavage sites, there is a dearth of computational tools to automate analysis of *in vitro* and *in vivo* protease assays. This necessitates individual researchers to develop their own analytical pipelines, resulting in a lack of standardization across the field. To facilitate protease research, here we present Protease Activity Analysis (PAA), a toolkit for the preprocessing, visualization, machine learning analysis, and querying of protease activity data sets. PAA leverages a Python-based object-oriented implementation that provides a modular framework for streamlined analysis across three major components. First, PAA provides a facile framework to query data sets of synthetic peptide substrates and their cleavage susceptibilities across a diverse set of proteases. To complement the database functionality, PAA also includes tools for the automated analysis and visualization of user-input enzyme–substrate activity measurements generated through *in vitro* screens against synthetic peptide substrates. Finally, PAA supports a set of modular machine learning functions to analyze *in vivo* protease activity signatures that are generated by activity-based sensors. Overall, PAA offers the protease community a breadth of computational tools to streamline research, taking a step toward standardizing data analysis across the field and in chemical biology and biochemistry at large.

## Introduction

Proteases play essential roles in diverse biological processes ranging from development to differentiation, and dysregulated protease activity is a driver of a variety of pathological conditions including cancer, neurodegeneration, and infectious diseases.^1^ Because proteases most proximally exert their function through their *activity*, understanding protease activity, rather than transcript or protein expression, is required to elucidate the biological roles of proteases and to harness these enzymes as diagnostic and therapeutic targets. To this end, molecular tools such as activity-based probes (ABPs), short synthetic peptide substrates, and noninvasive enzyme activity sensors have been developed to measure protease activities *in vitro*, i.e., of recombinant proteases or enzymes present in biospecimens,^2−6^ as well as *in vivo*, i.e., within the disease microenvironment.^7−11^ Beyond their use as a discovery tool, sensors that quantify protease activity are being applied directly for early detection and monitoring of disease,^5,12−918^ biological imaging,^7,18^ and drug discovery.^19,20^ Furthermore, proteolytic cleavage of peptide linkers is being used to trigger disease-specific activation of engineered activity-based diagnostics^11^ and therapeutics,^21−24^ all of which inherently rely on assessments of protease activity for their design and optimization. To support the development of these new activity-based tools and to advance the study of protease biology at large, a wide range of databases and algorithms have been created to better identify protease substrates and cleavage sites, providing a clear demonstration of how protease research can benefit from computational tools.^25−27^

Rapidly identifying, designing, and characterizing new peptide substrates and activity-based sensors remains a major bottleneck toward advancing these applications. This is due to the promiscuous nature of protease activity, the combinatorial number of synthetically accessible substrates, and the dearth of methods to automate protease activity analysis and substrate design. Current protease databases and analytic tools also focus exclusively on endogenous substrates and cleavage sites,^28−30^ despite the fact that synthetic activity-based sensors and large-scale libraries of synthetic peptides are now standard tools for measuring and quantifying protease activity *in vivo* and *in vitro*. Furthermore, the development of these experimental and molecular methods has not been accompanied by scalable, modular data analytic workflows. The creation of computational packages similar to what exists for genomics (e.g., Bioconductor) could enable the standardized analysis of data generated from both *in vitro* and *in vivo* protease-based experiments. This would greatly benefit researchers by accelerating experimental workflows, informing diagnostic and therapeutic design, and facilitating biological insight into protease dysregulation in disease.

**Figure 1:**
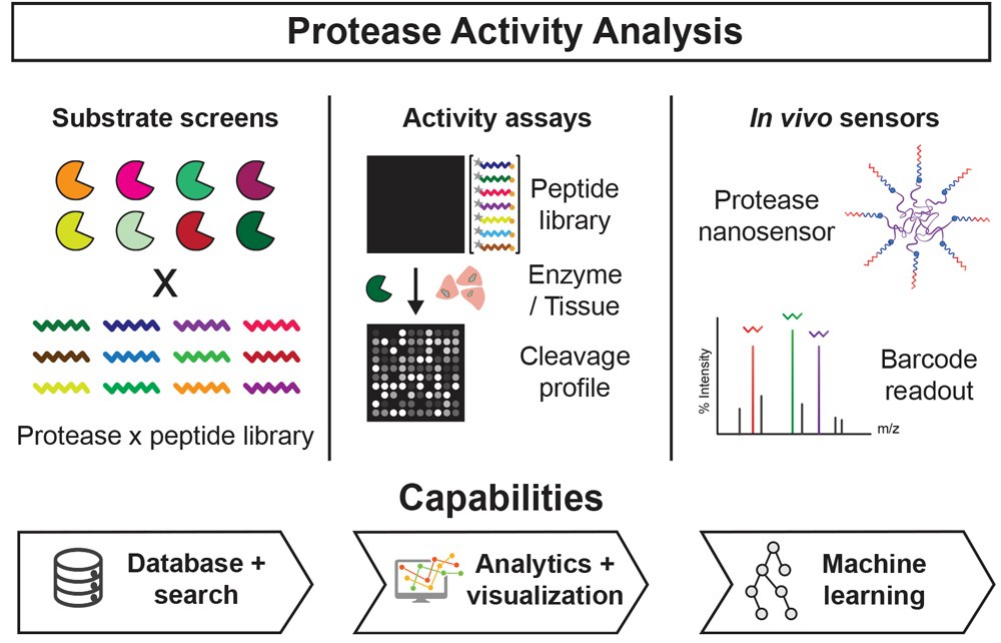
Overview of Protease Activity Analysis (PAA). The PAA package is designed to analyze data from large-scale substrate screens, enzyme activity assays, and *in vivo* enzyme activity sensors. Key package capabilities include searchable databases, where users can both query preloaded protease-substrate data sets published as part of PAA or import new data sets privately for their own use; data analytics and visualization functions, for facile and automated analysis of protease activity data; and machine learning models, for classification analysis of activity-based sensor data.

To address these needs, we present Protease Activity Analysis (PAA), a toolkit that addresses the need for computational methods to accelerate data analysis of enzyme activity data in biochemistry, chemical biology, and bioengineering (Figure 1). PAA contains a searchable database of existing protease activity data, curated from over a decade of published works from our group, along with modular analytics that enable users to query these data sets for enzymes or substrates of interest. Through PAA’s framework, users can additionally create and search new databases using their own protease-substrate screening data. PAA enables analytic standardization via functions that automate the quantification and visualization of user-input data from *in vitro* protease activity screens and *in vivo* protease-activated nanosensors. The package is accompanied by step-by-step tutorials that detail the functionalities provided by PAA in an open-source repository (https://github.com/apsoleimany/protease_activity_analysis). PAA’s Python-based implementation provides a modular framework that is easy to interface with other software packages and can be readily integrated into broader data analytic workflows.

## Results

PAA provides scalable and modular analysis capabilities for data sets of enzyme activity measurements. Specifically, PAA supports three core analytic workflows (Figure 1): (1) analysis and query of databases of peptide substrate sequences and their cleavage susceptibilities; (2) analysis of substrate screens using recombinant enzymes or biospecimens; and (3) analysis of measurements from protease-responsive *in vivo* nanosensors. Across all three workflows, PAA provides preprocessing, visualization, machine learning, and search functionalities.

### PAA Supports Searchable Enzyme–Substrate Databases

Identifying and characterizing which substrates are robustly and specifically cleaved by proteases of interest, such as those that are overexpressed in a specific cancer, is critical to discovery and engineering efforts that seek to understand and exploit protease activity. Indeed, the rapid rise and promise of engineered conditionally activated diagnostics and therapeutics, which almost universally incorporate a protease-cleavable peptide linker as the “trigger” for disease-specific activation, has motivated the need for tools and methods that identify synthetic peptide substrates that are maximally cleaved in diseased tissues and/or by target proteases. To this end, in PAA we present a *SubstrateDatabase* data structure that provides a facile framework for curating and querying data sets of enzyme–substrate activity, which often take the form of fluorometric assays of proteolytic cleavage of synthetic, fluorogenic peptide substrates. These assays measure the kinetics of enzyme activity over time and can be used to assess both the efficiency of an enzyme for a particular substrate, by quantifying the initial rate of the reaction, as well as the specificity of a substrate for a protease, by comparing the substrate’s cleavage against other proteases screened.

**Figure 2:**
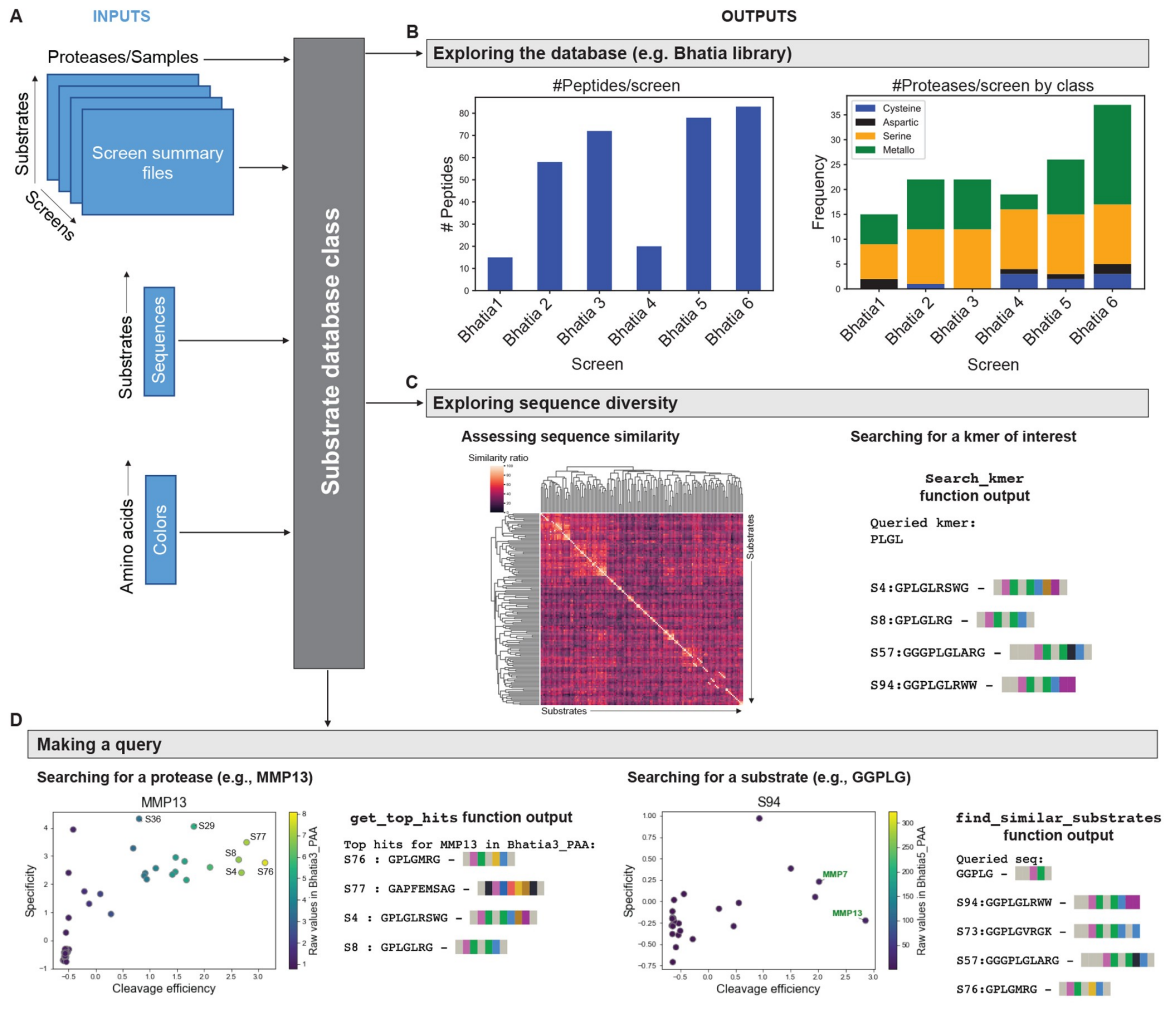
PAA provides an infrastructure for queryable databases of enzyme substrates. (A) *In vitro* activity screen summary files, a substrate sequence file, and an amino acid color map file provide data inputs for a *SubstrateDatabase*. (B) Sample use of the *SubstrateDatabase* to query a database of 150 unique substrate sequences screened against a diverse set of recombinant proteases. Summary plots of number of substrates and proteases across six independent screens comprising the database. (C) Metrics of sequence diversity include hierarchical clustering of pairwise sequence similarity scores as well as the ability to search for *k*-mers of interest. (D) Sample outputs of querying the database for a protease of interest (e.g., MMP13) and a sequence or cleavage motif of interest (e.g., “GGPLG”).

To demonstrate these capabilities, we have created a publicly available database that incorporates data generated by our group from six independent recombinant protease screens against fluorogenic peptide substrates. The database consists of 150 unique synthetic peptide substrates and their cleavage susceptibilities across a set of 77 distinct recombinant proteases spanning the metallo, serine, cysteine, and aspartic catalytic classes. The substrates published as part of PAA were identified based on the literature and designed to query the activity of disease-associated proteases, including those in cancer, infection, and thrombosis. As such, there is an over-representation of metallo- and serine-sensitive substrates in the data set, which is open-sourced as a part of PAA (Figure 2B).^13,31^ However, users can also import their own data into PAA for individual use, instantiating *SubstrateDatabase* data structures that can be readily queried and analyzed.

A guide to the core analytic and visualization functions related to the database can be found at https://github.com/apsoleimany/protease_activity_analysis/tree/master/tutorials. This step-by-step guide showcases how to load and query the protease-substrate database that is published with this work. Briefly, to instantiate a *SubstrateDatabase*, the user inputs raw data matrices of activity measurements (i.e., *n* × *k* where *n* is the number of substrates screened, and *k* is the number of conditions, e.g., proteases, assayed) for screens to be included in the database; a file that maps substrate names or labels to their corresponding sequences; as well as an optional file that maps amino acids to different colors based on properties of interest (e.g., hydrophobicity, chemistry, and identity) (Figure 2A). The *SubstrateDatabase* object first identifies overlapping substrates or proteases across multiple screens and aggregates all the data available for each unique substrate and protease into one simple data structure. In this way, protease-substrate activity assay data for proteases, substrates, or sequences of interest can be easily and efficiently queried. For example, if a user wants to identify potential substrates for a specific protease, they can input the protease name, and PAA will output a ranked list of substrates predicted to be efficiently and specifically cleaved by the protease of interest. The predictive rankings output by PAA strongly align with empirical results (Figure S1), lending strength to the power of PAA to help optimize protease activity experiments using *in silico* methods. Similarly, given a substrate as the user query, PAA can identify proteases that have been shown to robustly and/or specifically cleave that substrate. Note that, for the public data set published as part of PAA, substrate names correspond to names assigned by our group for specific sequences. These names map to existing nomenclature from previously published works for easy reference.^12,13,31^

Despite the fact that PAA contains cleavage data for a large number of substrates, in many cases the user will have a query sequence of interest that is not already included in the database. In the absence of an exact match, the *SubstrateDatabase* can retrieve the top-*k* substrates most similar to the query sequence, as quantified by different sequence similarity metrics. PAA offers two different sequence similarity metrics: the Levenshtein distance similarity ratio and the partial ratio. The former is strictly based on the Levenshtein distance that can be computed as the minimum number of single-character edits (insertions, deletions, or substitutions) required to change one amino acid sequence into another. The partial ratio metric works similarly but instead takes the shortest sequence and compares it with all substrings of the same length. This partial ratio is particularly useful when two substrates contain the same amino acid cleavage motif (e.g., “PLG”) but are flanked by different spacers at the N- or C-terminus (e.g., “GG” or “GS” spacers), as they will still be assigned high similarity estimates. After calculating the similarity between the user’s input sequence, PAA returns the *k* most similar sequences and the values of the similarity metrics (Figure 2D).

Furthermore, the database also incorporates informative metrics on sequence diversity across substrates (Figure 2C). Such estimates can be very useful during library optimization to characterize the degree of redundancy and orthogonality between substrates in a given peptide library. Alternative metrics have been recently described by others to achieve similar goals.^32^ To this end, PAA incorporates the function *get_similarity_matrix* that performs hierarchical clustering of pairwise similarity scores between all substrate sequences in the database and affords a compact visualization of sequence diversity. In addition, the *search_kmer* function allows the user to readily find all substrates in the data set that contain a given *k*-mer motif of interest, such as the metalloprotease cleavage motif “PLGL” (Figure 2C). By integrating data from both of these analyses, PAA can help guide library optimization by allowing the user to make inferences about clusters of substrates that may have similar protease cleavage susceptibilities based on similarity scores and known cleavage motifs. Additionally, PAA can identify substrates that rank most distinct from others in the library and thus may be favored or disfavored based on the application at hand.

All recombinant proteases included in the database are of human origin. However, because overlapping cleavage sites have been identified between protease orthologs,^33^ we anticipate that the human sequences in the database will prove useful to researchers studying proteases in model organisms. To enable users to search for orthologous protease genes across species, PAA includes a function, *species_to_species*, that builds off of the comprehensive “Mammalian Degradome Database”^34^ to facilitate mapping of genes across species of interest (human, chimpanzee, mouse, and rat). For instance, the function will map the human protease “*MMP9*” to its mouse ortholog “*Mmp9*” while alerting the user that human “*GZMH*” does not have a mouse ortholog.^35^ Furthermore, while PAA features data from our group published as a resource and example for the *SubstrateDatabase* data structure, users can use PAA as a local package to analyze their own data sets, which will keep all data private to the user. Then, users can implement PAA’s functionalities and modular methods to analyze their private or internally generated data sets, as well as other data sets of interest.

### PAA Provides Modular Methods to Analyze *In Vitro* Substrate Screens

**Figure 3:**
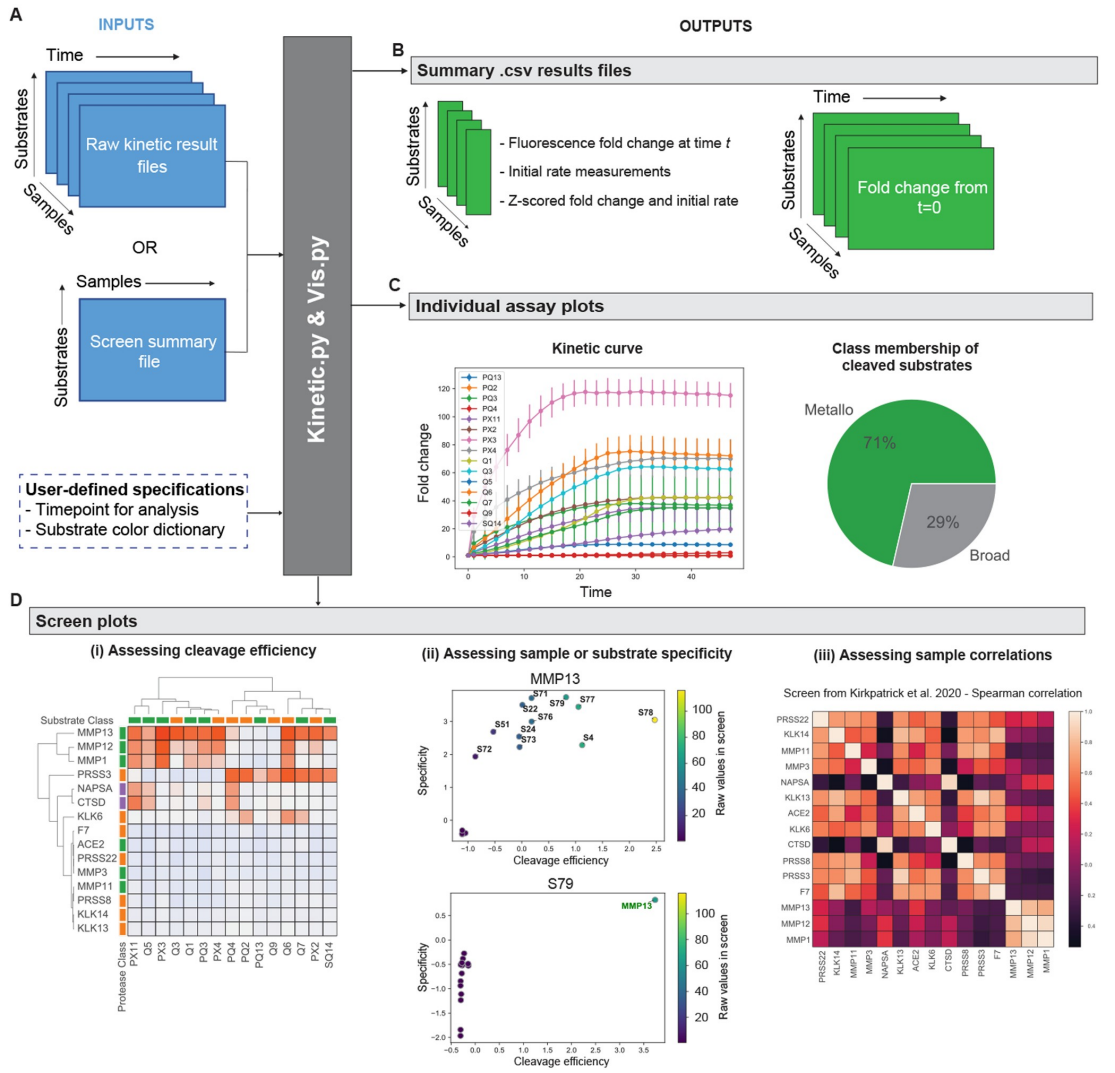
PAA automates analysis of *in vitro* assays of protease activity. (A) Fluorescence intensity measurements, together with user-defined specifications such as time points for analysis, are provided as inputs for construction of a *KineticDataset* . (B) *KineticDataset* automatically generates and saves key output activity measurements, such as initial activity rates and fold increases in substrate turnover as a function of time. (C) Retrospective analysis of 15 lung-cancer-associated proteases screened against a panel of 14 Forster resonance energy transfer (FRET)-paired substrates,^13^ with representative plots for MMP13 shown, including line plots of fold change intensity over time for each substrate and a pie chart summarizing substrate cleavage susceptibility. (D) For the same study,^13^ comprehensive assessment of cleavage efficiency and specificity across recombinant proteases and substrates. (i) Fluorescence fold changes were subject to hierarchical clustering to cluster proteases (vertical) by their substrate specificities and substrates (horizontal) by their protease specificities. (ii) Specificity versus efficiency (SvE) plots compare standard scores across substrates (efficiency; *x*-axis) against standard scores across proteases (specificity; *y*-axis). SvE analysis for the protease MMP13 shows promiscuous activity across substrates (top). SvE analysis for the substrate S79 highlights that it is specifically cleaved by MMP13 relative to other proteases assayed (bottom). (iii) Pairwise correlation analysis of initial rates across all substrates for recombinant proteases in the screen, measured as the Spearman rank correlation coefficient. Heatmap identifies the highest correlation of substrate cleavage between MMP1 and MMP12, among all proteases in the analyzed data set.^13^

As highlighted by the rich information that could be harnessed from PAA’s public data set of protease–substrate activity measurements (Figure 2), *in vitro* enzyme activity assays are vital to characterizing protease activities and their dysregulation in disease.^1,10,36^ Despite the broad prevalence of these assays throughout the protease biology and chemical biology communities, as well as the consideration that such assays will only become larger in size as high-throughput screening becomes more common, data analysis remains, to the best of our knowledge, largely manual. There is a dearth of computational tools to automate the analysis and visualization of enzyme activity data sets. PAA introduces a way to represent, store, and analyze these data sets automatically through the *KineticDataset* data object, which contains a suite of functions for rapidly preprocessing, visualizing, and analyzing these data (Figure 3).

The *KineticDataset* class is equipped to take in raw data files from enzyme activity assays (e.g., cleavage of fluorogenic substrates) generated directly by measurement instruments (e.g., fluorimeters; Figure 3A, Figure S2). Raw files consist of matrices of activity measurements for each sample to be analyzed (i.e., *n* × *t*, where *n* is the number of substrates screened and *t* is the number of time points recorded). The class automatically generates key output activity measurements, including initial rates (intensity/min^–1^) and fold changes at user-defined time points across substrates (Figure 3B). The resulting measurements can then be visualized with line plots that depict changes in raw fluorescence intensity and fold change intensity over time for each substrate (Figure 3C). Furthermore, users can define the catalytic class of each screened protease (e.g., metallo- versus serine-), to visualize the cleavage susceptibilities of their substrates by different protease classes (Figure 3C). This may prove useful if a certain protease class is known to be associated with a particular disease state, such as metalloproteases and cancer.

PAA also supports inputs from retrospective screens, for which a matrix summarizing cleavage efficiencies across a set of samples may have already been produced (Figure 3A). Based on these summary matrices, PAA can be used to cluster samples (e.g., recombinant enzymes, cell, or tissue lysates) of interest based on their activity patterns; to identify substrates that are cleaved with increased specificity by a given sample; and to examine correlations in cleavage patterns across screened samples (Figure 3D). In particular, specificity versus efficiency analyses (“SvE” plots) enable identification of optimal protease–substrate pairs from *in vitro* activity assays (Figure 3D). SvE plots are generated by calculating z-scores across the screened substrates, which serve as a surrogate metric for cleavage efficiency, and z-scores across the screened proteases, which serve as a surrogate metric for specificity. By plotting these metrics against each other, optimal substrate–protease pairs can be rapidly identified from large sets of screening data by identifying substrates that score high for both of these metrics (Figure 3D).^918^ In addition, annotation by the raw activity measurement values for each protease–substrate pair reflects the absolute cleavage rate of a substrate of interest and overcomes the relative nature of standard scoring. Altogether, these analyses enable rapid assessment of substrate cleavage efficiency and specificity as well as robust identification of differential or overlapping activity signatures across different enzymes or tissue types (Figure 3D).

A step-by-step tutorial of the core data input, processing, visualization, and analytic functions can be found at https://github.com/apsoleimany/protease_activity_analysis/tree/master/tutorials. The demonstrations and the results presented in Figure 3 feature a retrospective analysis of a previously reported *in vitro* screen of a panel of lung-cancer-associated proteases against a panel of 14 peptide substrates^13^ (Figure 3). We showcase the modularity of these functionalities through analysis of a second independent *in vitro* protease screen from the literature^32^ (Figure S2), demonstrating that PAA extends to data sets from different experimental setups and research groups.

Together, *KineticDataset* and the visualization functionality provided by PAA streamline the aggregation, visualization, and analysis of *in vitro* activity measurements. In particular, these analyses facilitate the assessment of cleavage efficiency and specificity as well as the identification of differential and overlapping activity signatures among different enzymes or tissue types.

### PAA Enables Machine Learning Analysis of *In Vivo* Activity Data

Because proteases play critical functional roles in a variety of disease processes, recent years have seen the emergence of new classes of activity-based diagnostics that are engineered to measure the activity of endogenous enzymes at the site of disease and to generate an output signal that can be read out externally.^11,37^ To this end, our group has developed activity-based nanosensors, probes that detect the activity of aberrant proteases within the body and generate exogenous urinary reporters that reflect the degree of proteolytic cleavage encountered *in vivo*.^12−14,16,918,38−42^ These nanosensors consist of an inert scaffold whose surface is decorated with peptide substrates, designed to be cleaved by proteases dysregulated in the disease state of interest. Each substrate is marked with a mass-encoded peptide barcode, which is released upon interaction of the nanosensor with target proteases and then concentrated in the urine. Upon collection of urine, the relative concentrations of each reporter are quantified using mass spectrometry. Multiple sensors can be multiplexed simultaneously by barcoding each unique peptide substrate with a different mass-encoded peptide reporter.^5,12−14,918,16,31^ This multiplexing generates *n* × *k* matrices of urinary reporter measurements, where *n* is the number of samples and *k* is the number of sensors/reporters. The reporters serve as input features, and thusPAA automates data analysis of these input matrices and enables training of downstream machine learning classifiers.

**Figure 4:**
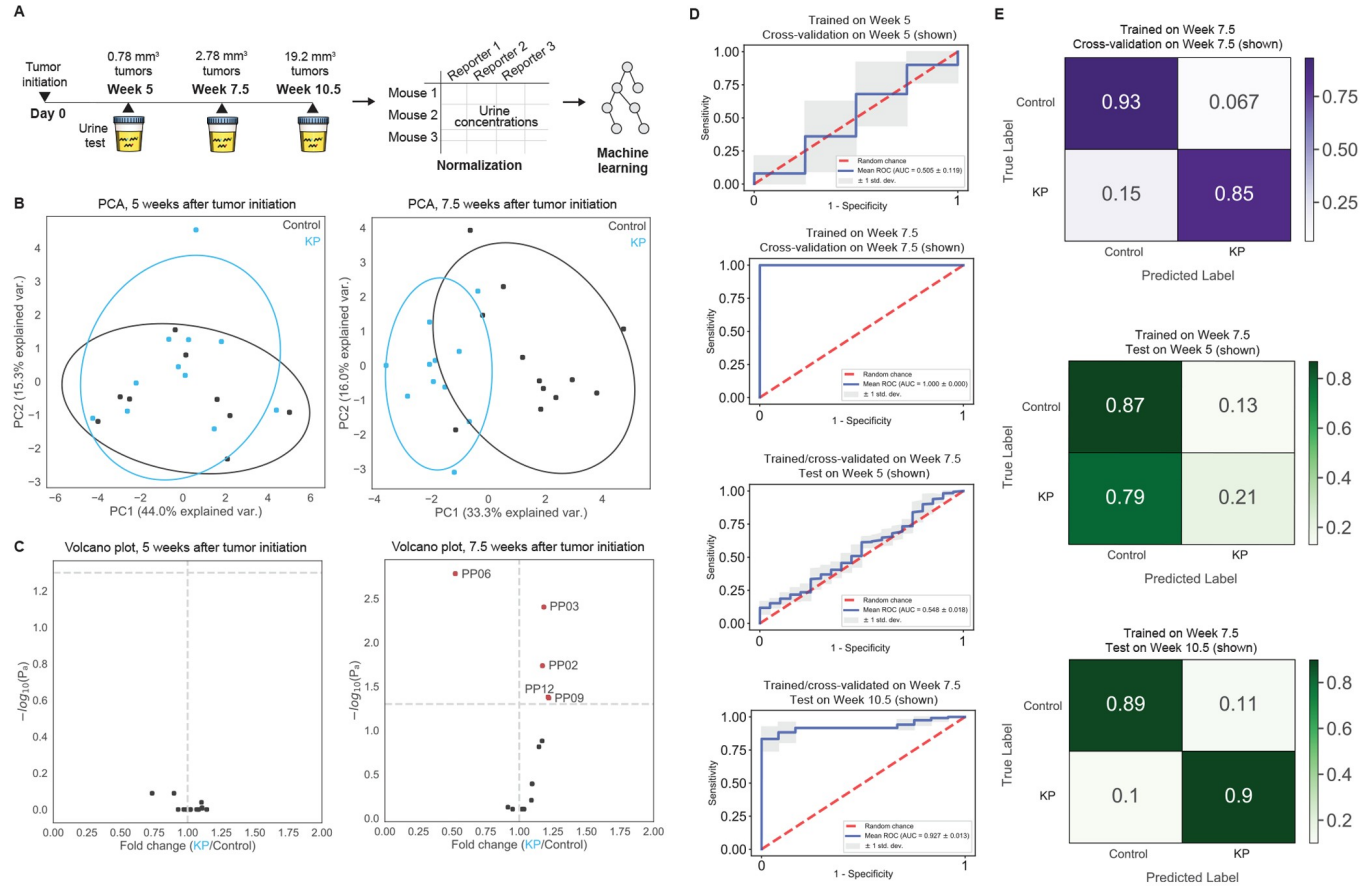
PAA enables automated machine learning analysis of *in vivo* activity data from activity-based nanosensors. (A) In a previously published study, activity-based nanosensors were administered at three different time points after tumor initiation in a mouse model of lung adenocarcinoma.^13^ Dysregulated protease activity in the cancerous lungs triggered the release of mass-encoded reporters into the urine. The urinary reporter concentrations were measured with mass spectrometry. PAA enables analysis, visualization, and machine learning on these data. (B,C) PAA automates analysis to visualize differences in reporter enrichment among conditions, such as different sample classes, e.g., wild-type control (Control) and lung cancer (KP) mice, and time points, e.g., 5 and 7.5 weeks after tumor initiation in KP mice. (B) Principal component analysis (PCA) can reduce the dimensionality of the feature space to discover differential activity signatures across conditions. (C) Volcano plots identify nanosensors driving these signatures, by comparing the fold change in reporter concentrations between two classes (*x*-axis) against their statistical significance (−log_10_(*P*_adj_); *y*-axis). (D) PAA evaluates the diagnostic potential of these activity signatures through automated training, validation, and testing of machine learning models, for example on the classification of healthy control and KP lung cancer mice. (E) Multiclass classifiers can also be trained, tested, and visualized using PAA.

Considering the flexibility afforded by this approach, we implemented a modular data framework, *SyneosDataset* for analysis and machine learning on these mass-barcoded reporter measurements. Our framework provides a variety of capabilities directly tied to biological, diagnostic, and analytic questions of interest. These capabilities include differential enrichment analysis of reporters between conditions (i.e., identifying which reporters are associated with disease versus healthy states); unsupervised dimensionality reduction; binary and multiclass classification; feature, sample, and data specification for all analyses; recursive feature elimination; and associated visualizations. To demonstrate these capabilities, we created a step-by-step guide, published as an implementation tutorial, that details input data requirements, analytic functions, and visualization options. This guide recapitulates the findings of previously published work demonstrating the noninvasive detection of localized lung cancer in mice using a 14-plex activity-based nanosensor panel.^13^ For the original study, the authors analyzed the *in vivo* data using unique scripts created specifically for their analysis. In our demonstration, we show how the same raw data can be parsed, analyzed, and visualized using the modular functions available in PAA (Figure 4). We developed modular machine learning functions that enabled the creation of additional diagnostic classifiers (Figure 4), thus demonstrating how PAA can be used to derive new insights from existing data.

Briefly, in the original study, a 14-plex nanosensor panel was administered into a mouse model of lung adenocarcinoma at 5, 7.5, and 10.5 weeks after tumor initiation and in parallel healthy controls (Figure 4A). After collecting the urine from each mouse, the urinary reporter concentrations were quantified using mass spectrometry. In the original study, the authors sought to determine the earliest stage at which the nanosensor panel could detect lung cancer. PAA streamlined normalization, statistical analysis, and machine learning of the nanosensor data into a single computational pipeline. We used this pipeline to verify that PAA’s modular workflow could recapitulate the original findings. PAA automated dimensionality reduction, specifically principal component analysis (PCA), to compare the urinary signals from each disease state across the tested time points (Figure 4B), and differential enrichment analysis to identify significant reporters (Figure 4C). In the example, one PCA plot (5 weeks after tumor initiation) shows an overlap between the two conditions, reflected in the volcano plot without any reporters being significantly differentially enriched (Figure 4B). In contrast, the PCA plot showing separation between clusters (7.5 weeks after tumor initiation) corresponds to differentially enriched reporters that drive the separation between groups, as reflected in the corresponding volcano plot (Figure 4C). With PAA, all graphs can be generated using a single function call in one line of code, making such analyses easily accessible, automated, and standardized.

The reporter concentrations can then be used to train binary and multiclass machine learning classifiers that can be used to diagnose disease (Figure 4D,E). PAA is capable of performing classification using a variety of algorithms, including support vector machines, random forests, and linear regression. This allows the user to benchmark methods rigorously and determine the best statistical learning method for their data. The user can also specify which sets of reporters, class labels, or individual labels should be used to train and test the classifiers. In the demonstration, we have shown that the reporter concentrations collected 5 weeks after tumor initiation are unable to yield a learned representation indicative of lung cancer, whereas by 7.5 weeks, the activity-based nanosensors generate an activity dataset that can be used to accurately diagnose lung cancer (Figure 4D,E). More generally, PAA provides a modular, streamlined data analytic workflow for measurements from *in vivo* protease nanosensors and can readily be applied to new data sets for automated statistical and machine learning analysis.

## Discussion

PAA advances computational methods to accelerate data analysis in biochemistry, chemical biology, and bioengineering. PAA represents a toolkit for users to automate the analysis of protease activity measurements generated *in vitro* through substrate screens or *in vivo* through noninvasive enzyme activity sensors. Here, we focus on the analysis of screens against synthetic, fluorescent-quenched peptide substrates (for the former) and of urinary reporter measurements from activity-based nanosensors (for the latter).

However, the modular methods and concepts presented in PAA readily extend to other data sets, particularly in terms of the tools for analysis of substrate screening data, as shown in Figure S2. Additional analytic functions for protease–substrate screening data, such as modeling time to cleavage saturation, prediction of competitive interactions between pairs of peptides,^43^ deconvolution of signals from mixtures of proteases,^44^ and identification of optimal substrate sets using principles from information theory,^32^ will expand PAA’s abilities to automate optimal enzyme substrate selection and design. Future work could extend PAA’s machine learning capabilities to include neural network models for classification analysis,^45^ methods for assessment of distribution shift and data set bias,^46−48^ as well as approaches for quantification of predictive confidence.^49−54^

Not only does PAA contain valuable analysis tools, but it also includes a publicly available database of 150 unique synthetic peptides and their cleavage susceptibilities across a set of 77 distinct recombinant proteases, together with an interface to query this database for proteases, substrates, or sequences of interest. PAA’s database can be readily expanded through publication and addition of new protease data, for example through high-throughput screening efforts that expand its coverage to additional enzymes. Users can use PAA as a local package to upload and analyze their own data sets, keeping all data private and leveraging PAA’s functionalities to query and analyze their data. PAA's modular database functionality and public dataset could be of great interest in the context of nomination of protease-cleavable peptide linkers, for example for protease-activated diagnostics and therapeutics. By focusing on synthetic substrates that directly measure protease activity and providing modular data science functionalities through an accessible software package, PAA’s database and analytic capabilities directly complement existing tools for assessing protease cleavage patterns.^28−30,44^ Being implemented and released as a Python package, PAA can be further developed and integrated into larger data analytic workflows. We envision that PAA will accelerate analysis workflows for biologists, biochemists, and engineers interested in understanding and leveraging protease activity to better understand, detect, and treat disease.

## Methods

PAA’s core relies on *NumPy*, *SciPy*, *Matplotlib*, *pandas*, *seaborn*, and *scikit-learn*. The Python-based implementation allows for flexible use, easy interfacing to machine learning and data analytic packages, and object-oriented programming. PAA's open-source code is available at https://github.com/apsoleimany/protease_activity_analysis and is published under the MIT license. PAA is organized and built as a package for ease of use and to facilitate developer integration.

The demonstrations described in this work are stored as Jupyter notebooks available in the PAA repository. These include: (1) querying and analysis of protease–substrate databases; (2) analysis and aggregation of *in vitro* screens of recombinant proteases and tissue lysates against synthetic peptide substrates; and (3) analysis and machine learning classification of urinary reporter signatures from *in vivo* activity-based nanosensors. The data sets used in these demonstrations were generated by our research group and are published together with PAA.

All code was written in the Python programming language. The PAA package is compatible with Mac OS, Windows, and Linux operating systems.

## Supporting Information Available

Code availability; Figure S1: Empirical validation of predictions for substrate ranking; Figure S2: Automated analysis of protease activity data from the literature (PDF)
