# Supplementary material for: Protease Activity Analysis: A Toolkit for Analyzing Enzyme Activity Data
Source: ACS Omega. 2022 Jul 6;7(28):24292–301. doi: 10.1021/acsomega.2c01559 (PMC9301967; doi:10.1021/acsomega.2c01559)
Supplement: Supplementary file 1 — ao2c01559_si_001.pdf [file ao2c01559_si_001.pdf]

Supporting Information

Protease Activity Analysis: A Toolkit for Analyzing Enzyme Activity Data

Ava P. Soleimany,[*,†,‡,¶,#] Carmen Martin-Alonso,[†,#] Melodi Anahtar,[†,#] Cathy S. Wang,[§] and Sangeeta N. Bhatia,[*,†,‖,⊥]

†*Harvard-MIT Division of Health Sciences and Technology, MIT, Cambridge, MA*

‡*Program in Biophysics, Harvard University, Boston, MA*

¶*Microsoft Research New England, Cambridge, MA*

§*Department of Biological Engineering, MIT, Cambridge, MA*

‖*Department of Electrical Engineering and Computer Science, MIT, Cambridge, MA*

⊥*Howard Hughes Medical Institute, Cambridge, MA*

#*Equal contribution.*

*E-mail: avasoleimany@microsoft.com; sbhatia@mit.edu

Supporting Information (3 pages)

Code availability; Figure S1: Empirical validation of predictions for substrate ranking; Figure S2: Automated analysis of protease activity data from the literature.

Code availability

All source code for the PAA package and step-by-step tutorials can be found at `https://github.com/apsoleimany/protease_activity_analysis`.

Additional Results

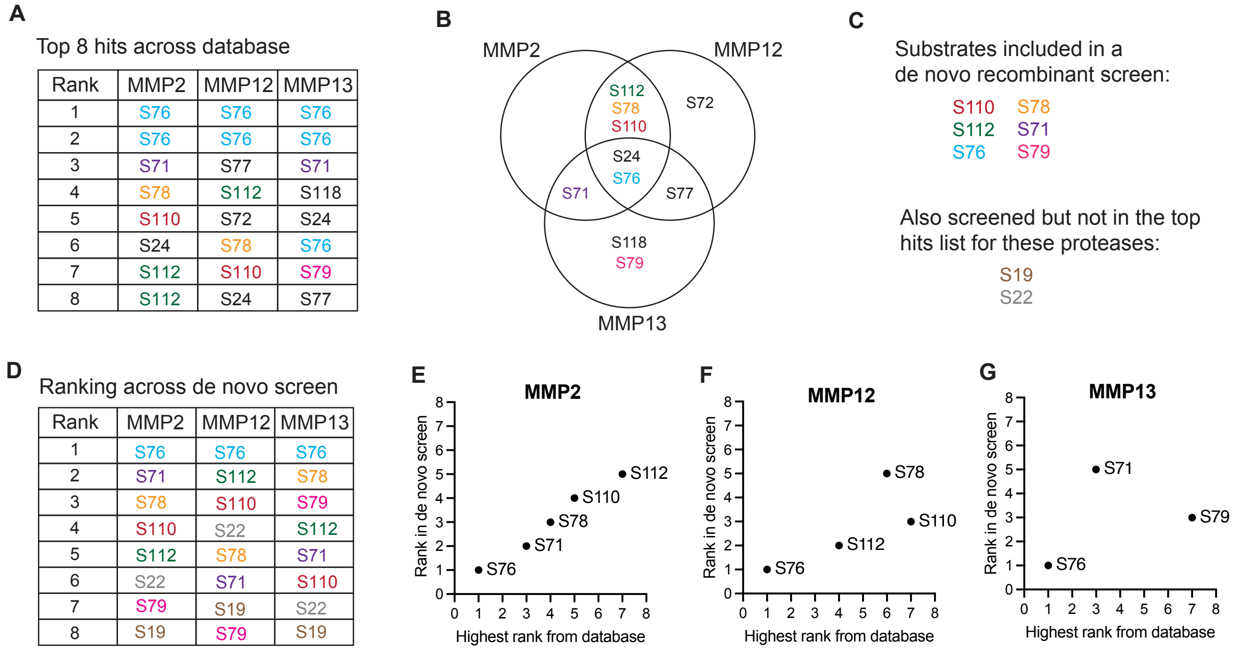

A Top 8 hits across database

Rank	MMP2	MMP12	MMP13
1	S76	S76	S76
2	S76	S76	S76
3	S71	S77	S71
4	S78	S112	S118
5	S110	S72	S24
6	S24	S78	S76
7	S112	S110	S79
8	S112	S24	S77

D Ranking across de novo screen

Rank	MMP2	MMP12	MMP13
1	S76	S76	S76
2	S71	S112	S78
3	S78	S110	S79
4	S110	S22	S112
5	S112	S78	S71
6	S22	S71	S110
7	S79	S19	S22
8	S19	S79	S19

Figure S1: **PAA predicts substrate rankings that are robust empirically.** **(A)** PAA's database was queried to obtain the top substrate hits for three MMPs: MMP2, MMP12, and MMP13. S76 was the top hit for all three MMMPs, but additional substrates ranked differently as top hits. **(B)** The three queried proteases belong to the same protease class, but only two of the top hits were shared between the three proteases. **(C)** To determine the robustness of the rankings output by PAA, a new *in vitro* screen of MMP2, 12, and 13 was run against 6 of the "top hit" substrates and two other substrates that did not make this list (S19 and S22). **(D)** The cleavage efficiencies of the 8 selected substrates by the 3 MMPs were ranked relative to one another. **(E-G)** To visualize differences between the empirical vs. predicted rankings, the highest predicted rank of each substrate from the database query was plotted against the empirical rank of the substrate in the independent *de novo* screen. A positive correlation corresponds to agreement between the predicted rankings and the empirical results.

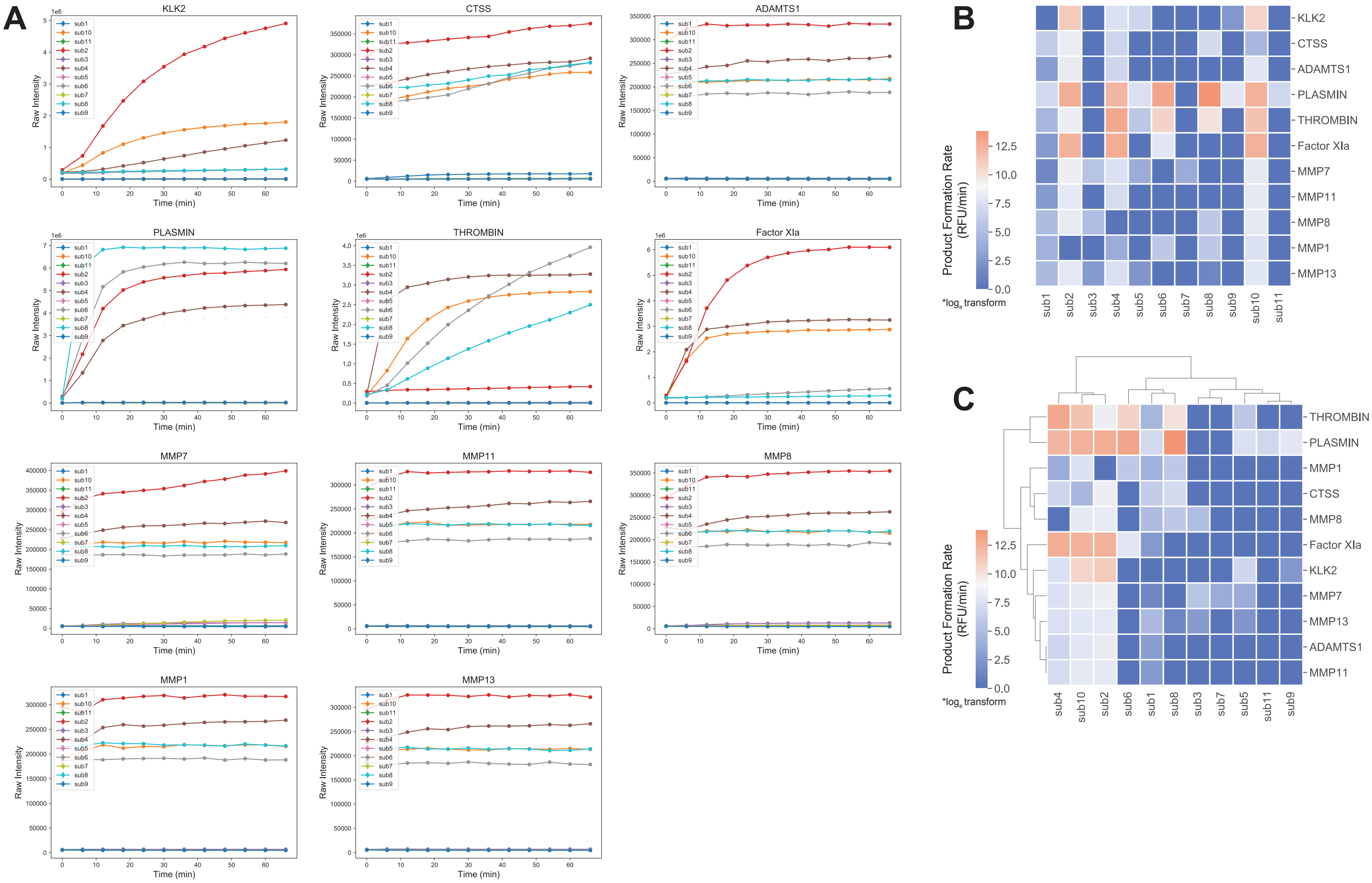

Figure S2: **PAA enables faithful, automated analysis of protease activity data from the literature.** Functions from PAA's KineticDataset were used to analyze kinetic substrate cleavage data from a protease-substrate *in vitro* screen from Holt and colleagues.[32] **(A)** PAA generates kinetic curves of raw fluorescence intensities as shown, for individual proteases screened against 11 substrates. Product formation rates are expressed in relative fluorescence units per unit time (RFU/min). **(B)** Heat map of product formation rates, calculated from kinetic activity curves in (A) and automatically generated by PAA. **(C)** PAA enables hierarchical clustering across proteases and substrates, rendering further insights into the relative cleavage similarities between proteases and substrates screened.
